# Quantitative assessment of endotracheal tube (ETT) biofilm by micro-CT scan: evaluation of the effectiveness of ETT cleaning devices

**DOI:** 10.1186/cc12094

**Published:** 2013-03-19

**Authors:** A Coppadoro, G Bellani, T Mauri, R Borsa, V Meroni, A Barletta, M Teggia Droghi, A Lucchini, R Marcolin, N Patroniti, S Bramati, A Pesenti

**Affiliations:** 1 University of Milan-Bicocca, Monza, Italy; 2 San Gerardo Hospital, Monza, Italy

## Introduction

The goal of this study was to assess the amount of biofilm within the endotracheal tube (ETT) by CT scan and to evaluate in a bench test a device aimed at ETT biofilm removal. Biofilm grows within the ETT soon after intubation, resulting in increased resistance to airflow. Biofilm detachment from the ETT can result in bacterial colonization of the distal airways. The use of an ETT cleaning device could effectively reduce the patient's airflow resistance burden. The quantification and distribution of biofilm within the ETT might help to evaluate ETT cleaning device effectiveness.

## Methods

We collected ETTs of 11 critically ill patients after extubation. Micro-CT scan (SkyScan 1176; Bruker, Belgium) was performed using a resolution of 35 μm. Axial sections of the 20 cm above the cuff were reconstructed, and the volume of secretions was assessed by a density criterion. Microbiological cultures of the ETT lavage fluid were then obtained. Patient's demographics and clinical data were recorded. In a different set of bench experiments, we injected 1 ml water-based polymer into new ETTs of different sizes. We measured resistance to airflow before and after using an ETT cleaning device (Airway Medix Closed Suction System; Biovo Technologies, Tel Aviv, Israel). We also obtained resistance values of intact ETTs as controls.

## Results

The studied ETTs remained in place for a median of 7 days (IQR range 4 to 15). The amount of secretions assessed by CT scan was 0.293 ± 0.290 ml (range 0.032 to 0.777 ml). Secretion volumes were not related to patient severity at admission (SAPS 2, P/F ratio) or days of intubation; an inverse correlation with patient's age was present (*P *= 0.032, *R*^2 ^= 0.46). Bacterial growth was present in 9/11 (82%) ETT fluids cultures and *Candida *spp. showed an elevated prevalence (6/11, 55%). In the bench tests, the cleaning device reduced resistance to airflow (difference before and after cleaning 5.5 (95% CI = 8.9 to 1.6) cmH_2_O/l/second, *P *= 0.006). After cleaning, resistance resulted higher than intact ETTs, although with a clinically negligible difference (difference 0.3 (95% CI = 0.2 to 0.6 cmH_2_O/l/second), *P *= 0.032).

## Conclusion

Micro-CT scan is a feasible and promising technique to assess secretions volume in ETTs after extubation. The use of an ETT cleaning device decreases resistance to airflow in bench tests; the effectiveness of such a device in the clinical setting could be properly assessed by post-extubation CT scan.

